# Heart rate prediction with contactless active assisted living technology: a smart home approach for older adults

**DOI:** 10.3389/frai.2023.1342427

**Published:** 2024-01-12

**Authors:** Kang Wang, Shi Cao, Jasleen Kaur, Moojan Ghafurian, Zahid Ahmad Butt, Plinio Morita

**Affiliations:** ^1^School of Public Health Sciences, University of Waterloo, Waterloo, ON, Canada; ^2^Department of Systems Design Engineering, University of Waterloo, Waterloo, ON, Canada; ^3^Centre for Digital Therapeutics, Techna Institute, University Health Network, Toronto, ON, Canada; ^4^Institute of Health Policy, Management, and Evaluation, University of Toronto, Toronto, ON, Canada

**Keywords:** active assisted living, smart home, heart rate monitoring, healthy aging, nonintrusive healthcare monitoring, aging in place, machine learning

## Abstract

**Background:**

As global demographics shift toward an aging population, monitoring their heart rate becomes essential, a key physiological metric for cardiovascular health. Traditional methods of heart rate monitoring are often invasive, while recent advancements in Active Assisted Living provide non-invasive alternatives. This study aims to evaluate a novel heart rate prediction method that utilizes contactless smart home technology coupled with machine learning techniques for older adults.

**Methods:**

The study was conducted in a residential environment equipped with various contactless smart home sensors. We recruited 40 participants, each of whom was instructed to perform 23 types of predefined daily living activities across five phases. Concurrently, heart rate data were collected through Empatica E4 wristband as the benchmark. Analysis of data involved five prominent machine learning models: Support Vector Regression, K-nearest neighbor, Random Forest, Decision Tree, and Multilayer Perceptron.

**Results:**

All machine learning models achieved commendable prediction performance, with an average Mean Absolute Error of 7.329. Particularly, Random Forest model outperformed the other models, achieving a Mean Absolute Error of 6.023 and a Scatter Index value of 9.72%. The Random Forest model also showed robust capabilities in capturing the relationship between individuals' daily living activities and their corresponding heart rate responses, with the highest *R*^2^ value of 0.782 observed during morning exercise activities. Environmental factors contribute the most to model prediction performance.

**Conclusions:**

The utilization of the proposed non-intrusive approach enabled an innovative method to observe heart rate fluctuations during different activities. The findings of this research have significant implications for public health. By predicting heart rate based on contactless smart home technologies for individuals' daily living activities, healthcare providers and public health agencies can gain a comprehensive understanding of an individual's cardiovascular health profile. This valuable information can inform the implementation of personalized interventions, preventive measures, and lifestyle modifications to mitigate the risk of cardiovascular diseases and improve overall health outcomes.

## 1 Introduction

### 1.1 The impact of aging population and the need for active assisted living

The world is currently experiencing a significant demographic shift, characterized by a rapid increase in the aging population. This demographic milestone is projected to reach unprecedented levels in the coming years (United Nations, 2019). Advancements in healthcare and living standards have led to an increase in the lifespan of individuals, resulting in a significant rise in the proportion of the older individuals within the global population (Quora, 2022). In 2022, the world wide population of individuals aged 65 years or older is more than 770 million, which means more than one in 10 people are older adults (Alvarez, 2023). Eastern and South-Eastern Asia are home to the largest number of older adults who are 65+ years old, accounting for ~260 million individuals, followed by Europe and Northern America with over 200 million older individuals, representing the highest percentage of older adults (more than 17%) (United Nations, 2019). These numbers are projected to increase over the next three decades, with an estimated one in six people worldwide being aged 65 years or over by 2030, and this figure is expected to double to 1.5 billion individuals by 2050. Especially in low and middle-income countries, where 80% of older adults are projected to reside, healthcare challenges may be significant [World Health Organization (WHO), 2023].

Chronic non-communicable diseases, including heart diseases, cancer, and chronic respiratory diseases, often accompany the process of aging (Prince et al., 2015). These conditions require specialized healthcare services that monitor various health indicators, including physical activity, heart rate, blood pressure, and sleep quality (Soon et al., 2019). Active Assisted Living (AAL) technology, i.e., technology that aims to maintain older adults' living independence using technology advancements, has emerged as a promising solution to address the healthcare needs of the aging population. By embedding reliable devices and sensors within a house, AAL creates a “smart home” environment (Domb, 2019). When coupled with Artificial Intelligence (AI), Smart Home Technologies (SHTs) has the potential to assist with daily functions in the home and monitor, treat and manage chronic health conditions (Philip and Williams, 2019). Moreover, smart homes can allow caregivers to provide better healthcare for older adults and even reduce their reliance on caregivers (Frisardi and Imbimbo, 2011). Smart home devices can continuously capture health-related information in an unobtrusive manner, thereby enabling safer independent living for the older adults (Wang et al., 2021). The integration of SHTs with AI offers the potential to constantly observe, model, and understand human behavior, as well as identify early warnings for interventions (Chen et al., 2014). Compared to other Internet of Things (IoT) technologies, such as wearables, SHTs do not require older individuals to carry it all day, which can be inconvenient or easily forgotten, particularly for those individuals living with dementia (Stavropoulos et al., 2021).

### 1.2 Heart rate prediction and its importance in cardiovascular health monitoring

Cardiovascular diseases (CVDs), also known as heart-related diseases, stand as the leading cause of death worldwide. In 2019 alone, CVDs accounted for 17.9 million deaths, representing 32% of all global deaths (World Health Organization, 2021). These devastating consequences are particularly prevalent in low- and middle-income countries, where limited access to healthcare services hinders early detection and treatment (Wirtz et al., 2016). As individuals age, the prevalence of CVDs increases, with the aging population being more susceptible to these diseases compared to younger individuals (Rodgers et al., 2019). A report from Benjamin et al. (2019) indicated that 40% of individuals aged between 40 and 59 have CVDs, while more than 70% of older adults aged 60 and above were affected by CVDs. Heart Rate (HR) serves as a key risk factor for CVDs and has been found to have a strong positive association with cardiovascular morbidity and mortality (Saxena et al., 2013). Consequently, accurate and timely prediction of heart rate can greatly contribute to the early detection and prevention of cardiovascular events, thereby improving overall cardiovascular health outcomes.

### 1.3 Current challenges and objectives

Despite the significant potential of SHTs and AI in predicting heart rate and enhancing cardiovascular health monitoring, there are several challenges that need to be addressed. Existing literature primarily focuses on wearable devices and IoT technologies for health monitoring, which often rely on user compliance and can be burdensome for older adults (Mitratza et al., 2022). Furthermore, the application of SHTs in predicting heart rate has been limited to specific contexts or necessitates the combination of wearable technologies, rendering it impractical for daily living scenarios. Therefore, the objective of this research paper is to investigate the feasibility of utilizing SHTs, coupled with AI, for heart rate prediction in an unobtrusive manner within an active assisted living environment. The paper aims to bridge the existing gap in research and contribute to the development of a comprehensive and practical solution for predictive monitoring of heart rate in real-world scenarios, addressing the limitations of wearable devices and current monitoring approaches. The objectives assume that smart home indicators, such as motion sensors and power consumption, can observe activity patterns and their correlation with changes in HR and studies whether such correlation exists.

### 1.4 Overview and paper structure

The increasing prevalence of cardiovascular diseases among the aging population highlights the urgent need for reliable indoor heart rate monitoring. This study leverages the potential of smart home technologies to address the need, proposing a contactless, machine learning-based solution for heart rate prediction. This paper is organized as follows: Section 1, the Introduction, introduces the topic and outlines the current challenges in the field. Section 2, Related Work, reviews relevant literature to provide an overview of the state-of-the-art in using smart home technology for heart rate prediction. Section 3, Materials and Methods, describes the devices used for the study, along with the deployment of the smart home environment. This section also details the methodology employed in the study. Section 4 presents the Results and Findings, where the analyses performed are reported. Finally, Section 5 discusses the implications of these findings, acknowledging the limitations of the current study and suggesting potential areas for future research.

## 2 Related work

This section presents an overview of previous research conducted in the field of heart rate prediction using IoT technology, machine learning, and smart home approaches for older adults. Lin et al. (2021) have explored the use of IoT technology to monitor cardiovascular events. They provided a comprehensive overview of sensor advancements capable of monitoring various physiological signals associated with CVDs. However, most of these sensors require attachment to the body. To address the limitations associated with inconvenience to carry of traditional health monitoring devices, Xiao et al. (2020) developed an intelligent wearable bracelet that can monitor heart rate during sports and provide real-time alerts for abnormal records. Similarly, David Chung Hu et al. (2018) proposed an intelligent monitoring system by integrating temperature sensors, pulse sensors, and accelerometers to measure the vital signs in older adults. The system demonstrated high accuracies, exceeding 90%, in measuring body temperature, pulse rate, and fall detection. Through the comparison of pulse rates during various activities, the study revealed substantial variations in heart rate across different activities. Ali et al. (2019) developed a wearable sensor prototype for heart rate and body temperature monitoring, achieving a low deviation of 2% compared to commercially available devices. Al Rasyid et al. (2015) integrated body temperature senor and pulse oximeter sensor into a wireless body sensor network to monitor various vital signs. This study identified a limitation related to the distance threshold (20 m) between the body-attached sensors and the server receivers, and the greater distance, the more data loss. Alnaggar et al. (2023) utilized Eulerian Video Magnification for non-contact heart rate and respiration rate extraction from video signals using camera. Although the proposed architecture are effective in extracting heart rate estimation from facial recordings, they face limitations with high-frequency noise, varying light conditions, motion, and raise privacy concerns, making it less suitable for daily living monitoring. The integration of AI techniques enables researchers to gain deeper insights from heart-related data and predict cardiovascular events. Dami and Yahaghizadeh (2021) proposed a deep learning method called LSTM-DBN, combining Long Short-Term Memory (LSTM) and Deep Belief Network (DBN) to detect arterial events with superior performance compared to other methods, such as Logistic Regression, Random Forest (RF), and Recurrent Neural Network. Mohan et al. (2019) introduced a hybrid machine learning model, HRFLM, which combined RF and Linear Model to predict cardiovascular diseases with a high accuracy of 88.7%. Wang and Gao (2020) developed a real-time heart rate monitoring system for athletes using wearable devices and deep learning techniques, achieving accurate heart rate prediction and activity classification in volleyball plays. Additionally, IoT based SHTs have been widely utilized in indoor healthcare and assisting older adults in aging in place (Linkous et al., 2019). Adib et al. (2015) utilized ubiquitous sensing technologies to remotely monitor vital signs using a wireless sensing technology called Vital-Radio, achieving a high accuracy of 99% for breathing and heart rate measurements. However, the performance of this method is limited by large body movements, as it primarily relies on detecting chest motion. In studies, Kumar et al. (2022) and (Liu et al. (2022), the authors utilized Wi-Fi technology to monitor vital signs by detecting physiological movements that affect channel state information. However, this technology also has limitations in accurately detecting high heart rates or breathing rates during periods of excessive movement. Scalise et al. (2016) developed a smart home network with heterogeneous health sensors to monitor cardiovascular situations in older adults, while Pham et al. (2018) presented a cloud based smart home environment for comprehensive homecare services for older adults.

Despite the advantages of wearable devices in terms of accuracy and non-invasiveness, challenges related to their adoption and user compliance exist. Wearable devices heavily rely on participants' willingness to wear them, and some individuals such as older adults with dementia may forget to wear them regularly, leading to incomplete data and reduced healthcare monitoring quality (Stavropoulos et al., 2021). Additionally, concerns regarding the use of unfamiliar devices in research studies and the discomfort associated with wearing them for extended periods can create resistance among users, especially older adults. Sensor burden and user discomfort have been identified as critical issues that need to be addressed in wearable sensor monitoring solution (Zhang et al., 2022).

However, current research exhibits a gap in the ability to monitor heart rate information using smart home technologies alone, without the need for additional wearable devices, which is more practical for daily living contexts. The primary contribution of this research is achieving precise heart rate prediction and enabling cardiovascular healthcare monitoring for different activities within the context of daily living without requiring additional involvement from individuals.

The scientific novelty of this study is demonstrating the adaptability of machine learning techniques to heterogeneous smart home indicators for indoor heart rate monitoring. Furthermore, the application novelty lies in exploring the potential of smart home technology to unobtrusively monitor heart rate for general surveillance, addressing the limitations of existing approaches.

## 3 Materials and methods

### 3.1 Devices

#### 3.1.1 AAL devices

Through the collaboration with smart home manufacturer Swidget, this study can acquire diverse smart home sensors and have access to their data repository through Application Programming Interfaces (APIs). The Swidget concept focuses on the adaptability and integration of smart home technologies. Their smart devices operate based on installing modular sensors into reconfigured common electrical devices such as outlets, on-off switches, and dimmers, which makes these devices unobtrusive, ensuring zero-effort from users. Moreover, these sensors are integrated directly into electrical circuits, meaning they do not have batteries. This ensures that they can be powered reliably for an extended period of time, without the concern of depleting battery life, provided there are no power outages. In this study, we paired smart outlets and smart switches with (1) air quality module sensors, (2) temperature and humidity sensors, and (3) passive infrared motion module sensors, which create four configurations in total. In addition, door contact sensors were also installed in the environment.

#### 3.1.2 Wearable devices

The purpose of using wearable device here was to collect the HR data, serving it as the benchmark when comparing with the predicted HR values. Consequently, it requires that the selected wearable device should be reliable and accurate. Empatica E4 wristband was selected as the HR measuring tool. Its accuracy has been validated by the study from Schuurmans et al. (2020), which demonstrated that Empatica E4 is a practical and reliable device for collecting HR data. Empatica E4 can measure the HR value with a sample rate of 1 HZ. In addition to HR, Empatica E4 is also equipped with an electrode, a photoplethysmography, a skin temperature, and a three-axis accelerometer, from which electrodermal activity, blood volume pulse, and skin temperature can be measured. Through the Empatica Manager application, Comma-Separated Values (CSV) files containing raw HR data can be downloaded from the devices.

### 3.2 AAL environment

**Figure 1:**
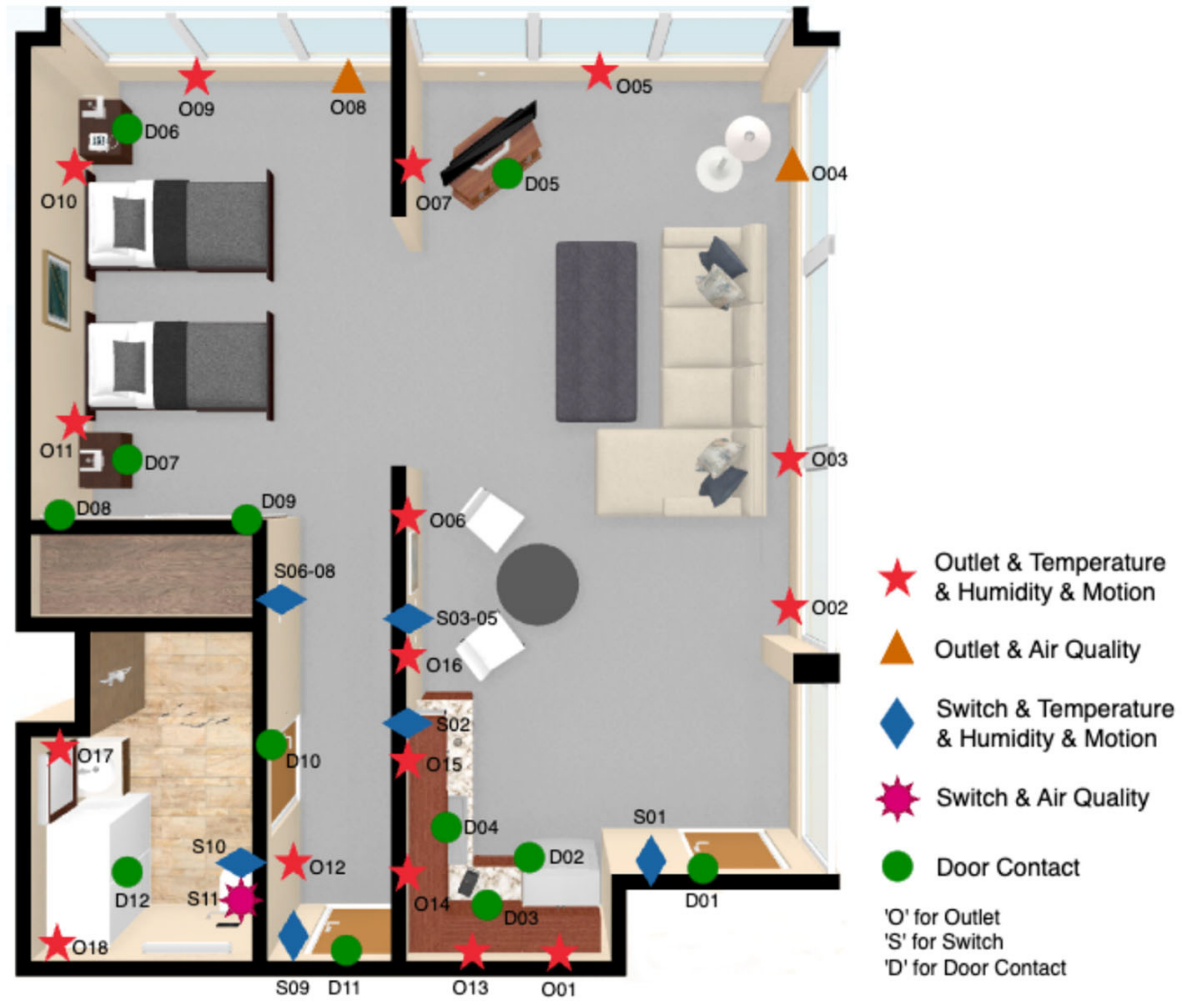
AAL environment.

A smart home environment was constructed in an apartment unit in the Research Institution for Aging in Waterloo (see Figure 1), with each device being assigned an ID. This apartment unit is composed of a kitchen, a living room, a bedroom, and a bathroom, which is decorated with necessary furniture to fulfill individuals' daily living requirements. The smart home environment was designed to be a sensor-rich space aiming to capture as many Activities of Daily Living (ADLs) as possible. According to locations of electrical devices in the room, we replaced original switches and outlets with Swidget intelligent devices and paired them with four types of environmental sensors.

When placing motion sensors in the environment, we followed the optimized sensor placement principles stated in Nasrollahzadeh et al. (2021), considering the detection range of Swidget motion sensor is 3 m, including (1) coverage: making sure the sensor has a wide enough field of view to detect movement in the area we want to monitor; (2) placement: mounting the sensor at a height of around seven feet to ensure it can detect movement across a room; (3) false alarms: positioning the sensor away from areas that is covered by furniture, such as behind sofa or bed, but areas where movement is likely to occur naturally to reduce false alarms. Door contact sensors were attached on each door, closet, and drawer to capture open/close events. Similarly, to get more accurate and comprehensive environmental data, we placed multiple temperature & humidity sensors and air quality sensors in different rooms. Additionally, we placed more sensors in the areas where these parameters are more likely to change, such as the kitchen, and the bathroom.

In order to better capture the ADLs happening in the room, we recruited five volunteers during the design phase to perform potential activities in the room, assisting us to iteratively test and adjust the locations and numbers of sensors.

### 3.3 Study

#### 3.3.1 ADLs scenarios

The ADLs scripts in this study was generated by following the principles in Edemekong et al. (2022). They cover basic ADLs and instrumental ADLs as well as single activities, posture-oriented, and movement-oriented activities, involving all spaces in the room, which can be representative for an individual's ADLs that happen within days. Each orientation requires different efforts from individuals and under each of them, we developed four to five scenarios to ensure the coverage of comprehensive ADLs.

Box 1 indicates that the activities designed for the different scenarios share similarities but different levels of activities, which can cause changes in heart rate. By repeating similar activities, a larger and more diverse dataset can be collected, which aids the machine learning model in distinguishing between various activities and heart rate patterns, thereby improving the robustness and accuracy of the models.

Box 1 **ADLs scenarios.**

1. Phase 1. Single activities

  • Scenario 1. Arriving home

 • Scenario 2. Eating dinner

  • Scenario 3. Sleep

  • Scenario 4. Cooking

  • Scenario 5. Going out

2. Phase 2. Posture-oriented

• Scenario 1. Reading on sofa

  • Scenario 2. Completing a puzzle

  • Scenario 3. Reading on bed

  • Scenario 4. Meal time

  • Scenario 5. Bathroom job

3. Phase 3. Movement-oriented

• Scenario 1. Tidying a room

  • Scenario 2. Morning exercise

  • Scenario 3. Taking shower

  • Scenario 4. Wandering in rooms

4. Phase 4. Basic ADLs

• Scenario 1. Cleaning rooms

  • Scenario 2. Changing footwear

  • Scenario 3. Prepare meal

  • Scenario 4. Make up

5. Phase 5. Instrumental ADLs

• Scenario 1. Arriving home from groceries

  • Scenario 2. Cleaning kitchen

  • Scenario 3. Emptying garbage

  • Scenario 4. Morning routine

  • Scenario 5. Wrap up things

#### 3.3.2 Participants

Recruiting participants for this study posed several challenges unique to the COVID-19 pandemic, especially with respect to our target population of older adults who are more vulnerable to infection. Consequently, we recruited students from the University of Waterloo as participants using convenience sampling. A total of 40 participants took part in the study, comprising 17 males and 23 females. The participants' mean age was 24 years, ranging from 18 to 39 years. Written informed consent was obtained from all participants before they took part in the study. To safeguard their privacy and confidentiality, participant information was de-identified. In order to minimize the bias arising from this age group difference, we instructed participants to simulate being seniors and engage in activities at a slower pace than their usual speed. The ethics of the study were reviewed and approved by the Research Ethics Office of the University of Waterloo under the ORE #43843.

#### 3.3.3 Procedure and data collection

Prior to the experiment, participants were asked to provide their informed consent and complete a questionnaire that was designed to collect demographic information. Subsequently, the researchers escorted participants to the smart home environment, where they were oriented to the locations of labeled sensors, appliances, and furniture. Additionally, participants were requested to wear Empatica E4 wristbands to generate benchmark heart rate data.

During the experiment, facilitators observed participants' activity performance through cameras in the room next to the apartment unit while delivering activity instructions to participants via a phone. Activity instructions were delivered in sequence by phases and scenarios to participant and no further instructions were provided unless participants asked for specific assistance. One minute transition time was allotted between each scenario, in order to reset all activated sensors. An observer documented activity information, including start and end timestamps, using an annotation sheet. Sensor readings were collected, formatted, and stored in the cloud database via our smart home data ecosystem. Only one participant was tested at a time, and each experiment session lasted ~3 h.

#### 3.3.4 Data pre-processing and summary

After performing data cleaning and excluding datasets from incomplete participants, a total of 28 participants' data remained available for further analysis. The data collected from the participants originated from two distinct sources, each with its own sampling rate. The smart home data gathered through 41 sensors shown in Figure 1 had a granularity of 30 s, while the HR data was recorded by the Empatica wristband at a rate of one measure per second. There is no missing data in the dataset, thereby eliminating the need for data imputation.

In the preprocessing stage for smart home data, we integrated data from different smart home sensors into a unified dataset. This integration involved structuring parameters from each sensor into individual columns within a single dataset. Consequently, each column in this dataset corresponds to a specific sensor parameter, facilitating ease of analysis and interpretation. Moreover, we ensured the synchronization of data across different sensors, leveraging the uniform timestamps and intervals recorded by each device. The synchronization process involved aligning data points from different sensors that shared the same timestamp.

To ensure compatibility between these datasets for HR prediction tasks, a methodical preprocessing approach was implemented for HR dataset, beginning with the synchronization of their timestamps. Let *t*_*home*_ represent the starting timestamp of the smart home dataset, and *t*_*HR*_ represent the corresponding starting timestamp in the Empatica dataset. The alignment process involves matching these starting points such that *t*_*home*_ = *t*_*HR*_. Following this synchronization, the HR data was resampled to align with the 30-s granularity of the smart home data. This was achieved by calculating the average HR over every 30-s intervals, starting from *t*_*HR*_. So for each 30-s interval in the smart home dataset, the corresponding ${\overset{\bar{\;}}{HR}}_{30s}$ computed, ensuring that the HR data is in sync with the smart home data.

**Figure 2:**
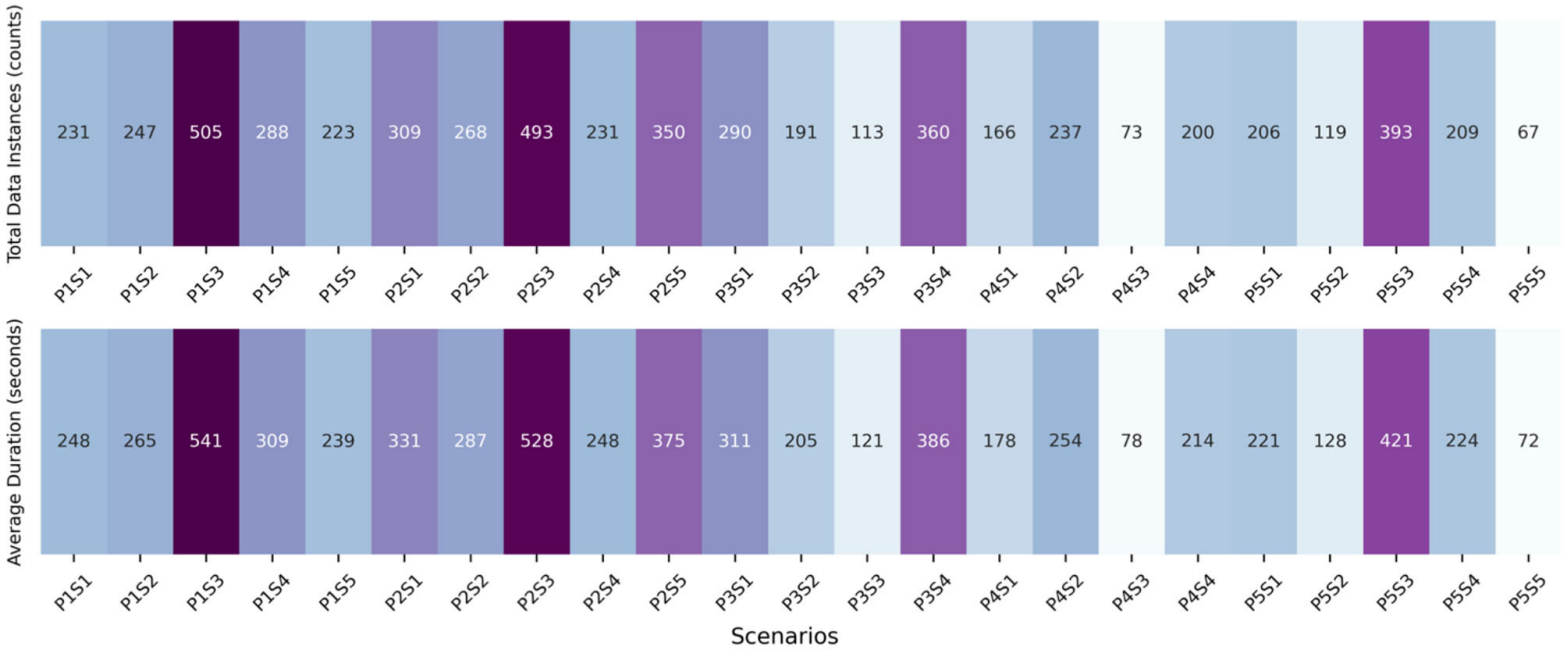
Total number of data instances and average time duration for each scenario.

In this study, each sensor recorded data for various features (see Appendix 1) during the experiment session. To ensure the accuracy and reliability of the data, we incorporated a 1-min transit time between each experimental scenario. It is essential to clarify that data from these transition times were not included in our analysis. During these transit periods, participants were inactive, allowing the sensors to reset to their initial state. This approach was crucial to mitigate any potential carryover effects from one scenario to the next. Consequently, it resulted in ~270 data instances per parameter for each participant. These instances represent a time series within each scenario, with each data point corresponding to a 30-s interval. However, it should be noted that these time series are not continuous between scenarios because of transition time. When aggregating data across all subjects, the final dataset comprised over 7,500 sample points. Figure 2 illustrates the total number of data instances collected from all subjects for each scenario, along with the average time duration (s) spent by subjects in each scenario.

Additionally, in the feature extraction process, we utilized parameters from each sensor, acquired through APIs at 30-s intervals, as individual features. This approach means that each sensor parameter was treated as a distinct feature. Specifically, for extracting temperature and humidity feature, we divided the environment into five separate rooms and extracted these parameters independently for each room. We selected sensors located in each specific room, and the room's temperature and humidity features were determined by averaging the readings from these sensors. Consequently, each participant's dataset comprised 137 features (Appendix 1) related to smart home indicators, and all of these features were utilized in the data analysis. These features encompassed a wide range of factors, including but not limited to motion occupancy, temperature, humidity, air quality, switch log, and energy consumption gather from each of the sensors in the environment. The inclusion of these smart home indicator-related features added depth and richness to the dataset, enabling a comprehensive analysis of the participants' living environment and activity patterns.

#### 3.3.5 Machine learning models and evaluation metrics

In this study, five classical machine learning models were selected to perform the prediction task. These models included support vector regression (SVR), K-nearest neighbor (KNN), RF, Decision tree (DT), and multilayer perceptron (MLP). Furthermore, to evaluate the performance of prediction models, several commonly-used metrics were used, which are root mean squared error (RMSE), mean absolute error (MAE), *R*-squared (*R*^2^), and scatter index (SI). These metrics comprehensively assess different aspects of model performance. Their equations are as follow:


$$RMSE\;=\;\sqrt{\frac{1}{n}\sum_{i=1}^{n}{\left({\hat{y}}_{i}\;-\;y_{i}\right)}^{2}}$$

$$MAE\;=\;\frac{1}{n}\sum_{i=1}^{n}\left|{\hat{y}}_{i}\;-\;y_{i}\right|$$

$$R^{2}\;=1-\;\frac{{\sum_{i=1}^{n}\left({\hat{y}}_{i}\;-\;y_{i}\right)}^{2}}{{\sum_{i=1}^{n}\left({\bar{y}}_{i}\;-\;y_{i}\right)}^{2}}$$

$$SI=\;\left(\frac{RMSE}{\frac{1}{n}\sum_{i=1}^{n}{\hat{y}}_{i}}\right)*\;100\%$$

where *y*_*i*_ is true value, $\bar{y_{i}}$ is the mean of true value $\hat{y_{i}}$ is the predicted value.

Specifically, RMSE calculates the square root of the average squared differences between predicted values and the actual values, while MAE measures the average absolute difference between predicted values and true values. *R*^2^ indicates how well the predicted values from the model fit the observed data, ranging from 0 to 1. SI provides a normalized measure of prediction error. A lower value of SI indicates better performance of the model. If the SI value is < 10%, it indicates a good model performance (Oyeleye et al., 2022).

#### 3.3.6 Evaluation setup

The data was process and analyzed on a Linux server (16GB RAM) with Ubuntu v22.04.3 operation system. Python v3.10.12 was used as the programming language to complete the data analysis task, along with its corresponding libraries such as Pandas v2.0.3, Matplotlib v3.5.1, and Numpy v1.21.5. Machine learning models were constructed based on the Scikit-learn v1.2.1 framework.

## 4 Results

### 4.1 HR data summary visualization

#### 4.1.1 Overall HR data distribution

**Figure 3:**
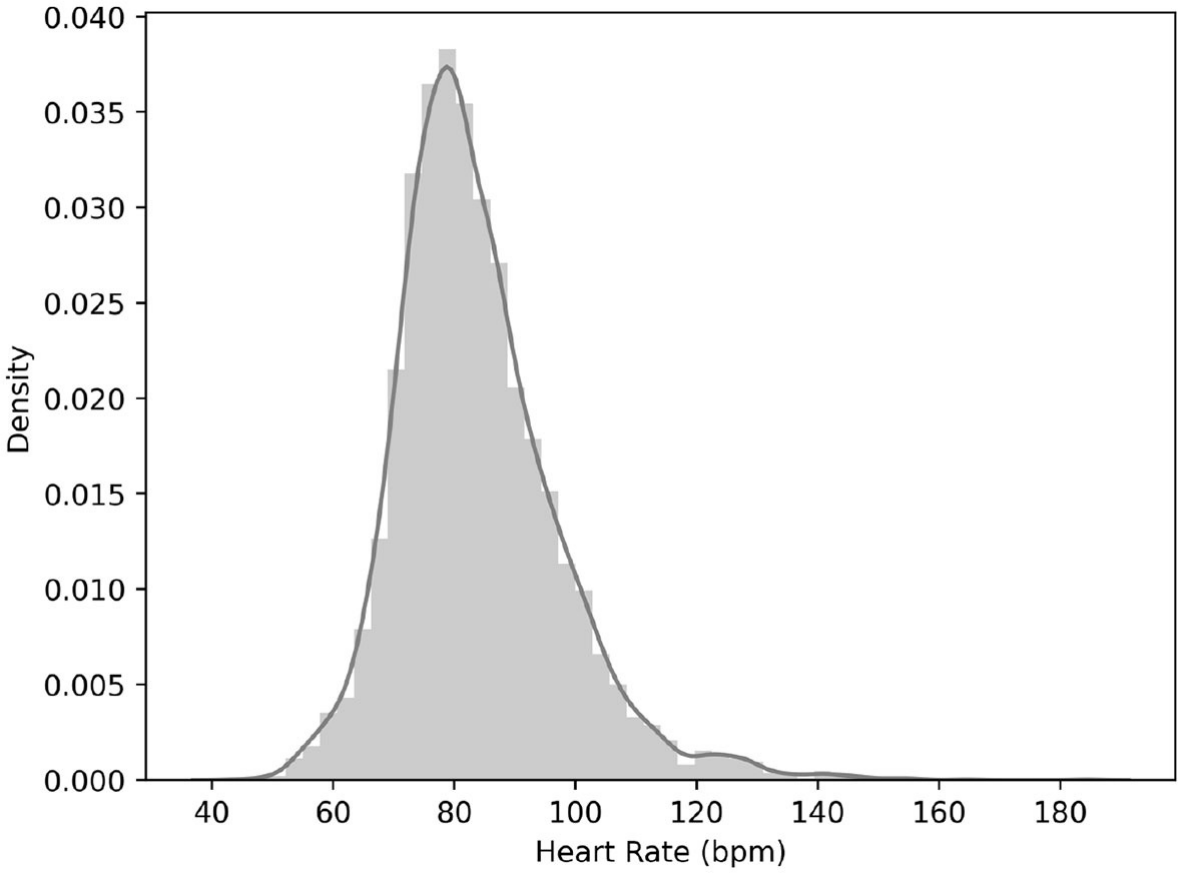
Heart rate distribution.

The distribution of heart rate data for all participants and activities is illustrated in Figure 3.

The majority of observations are concentrated around 80 beats per minute (bpm), which is commonly associated with typical resting heart rate. This suggests that a major portion of the data corresponds to individuals in a relaxed state, aligning with common indoor activities. A smaller proportion of instances is observed around 50 bpm, likely indicating periods of low activity intensity. The distribution of heart rate values also includes a proportion of samples ranging from 120 to 150 bpm, capturing instances of elevated heart rate during physical exercise scenarios.

This wide range of heart rate values reflects a spectrum of activity intensities within the set scenarios, from light to moderate and vigorous levels, highlighting the body's adaptive physiological responses to different forms of physical exertion. The distribution of the heart rate data appropriately reflects different activity intensities, providing a solid foundation for prediction analysis and ensuring the reliability and interpretability of prediction models (Yang et al., 2019).

#### 4.1.2 HR data distribution by scenarios

**Figure 4:**
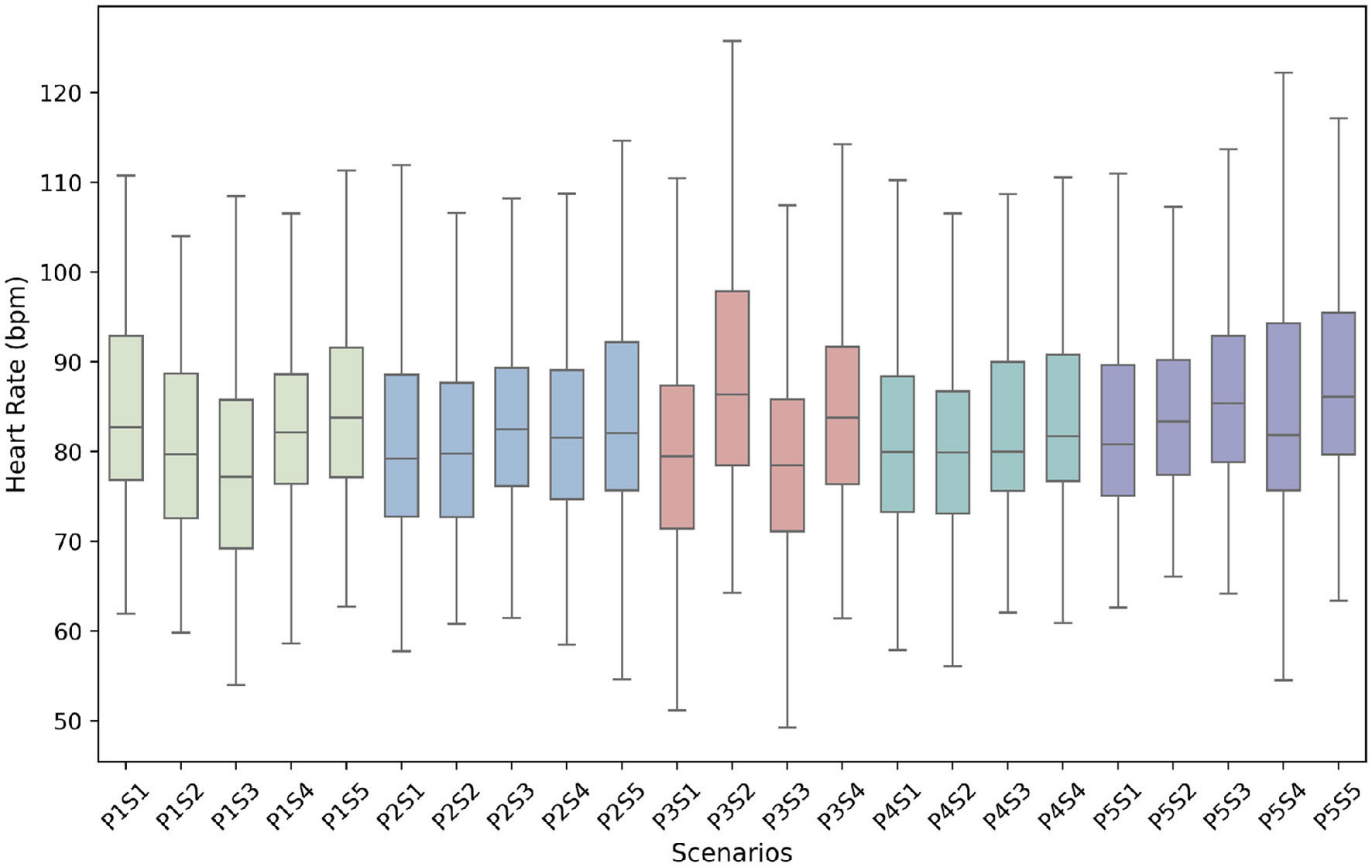
Heart rate data distribution on different scenarios (P stands for phase and S stands for scenario).

In this section, we analyzed the distribution of heart rate data in various scenarios (Figure 4) using boxplots to gain insights into physiological responses associated with different daily life scenarios. Each box represents a specific scenario, and the upper (or lower) whisker extends to the maximum (or minimum) data points within 1.5 times the height of the box from box top (or bottom). Data points outside of the whisker are considered as outliers, which have been excluded from the analysis. The thick line within the box represents the median, while the box itself represents the interquartile range between 25th and 75th percentiles, capturing the middles 50% of the data. This visualization provides insights into the spread or dispersion of the dataset.

The figure illustrates the heart rate data distributions across various scenarios, revealing some variation among them. However, the differences observed are not excessively pronounced and align with typical daily living situations. These fluctuations in heart rate are expected based on the nature of the performed activities. Phase 2, which comprises posture-oriented activities with minimal movement, exhibits a relatively stable data distribution across different activities. These activities typically involve low physical exertion, resulting in heart rates that remain within a relatively narrow range. The majority of heart rate data in Phase 2 falls between 70 and 90 bpm, indicating a consistent and moderate level of cardiovascular demand. Conversely, Phase 3 includes movement-oriented activities that require individuals to exert energy, resulting in considerable variability in heart rate data distribution across scenarios. The range of heart rate values for Phase 3 activities seems to be wider compared to other phases. Notably, the morning exercise scenario (P3S2) displays the highest heart rate distribution, with values exceeding 120 bpm, which may indicate the significant cardiovascular demands associated with this activity. Phase 1, encompassing a range of single activities including sleep, also exhibits noticeable variations in heart rate data. During sleep (participants only lied down rather than in actual sleep), heart rates tend to decrease due to the body's relaxation and decreased metabolic demands, resulting in the lowest recorded heart rate during this phase approaching ~50 bpm. On the other hand, Phases 4 and 5 show similar and consistent heart rate distributions. These distributions exhibit a notable right-skewed pattern, indicating that the majority of activities in these phases result in heart rates higher than the median value. This suggests increased cardiovascular demands during these activities. In other words, these activities generally involve more intense physical exertion, causing a higher cardiovascular response compared to the median level.

### 4.2 Heart rate prediction

#### 4.2.1 Different models

The whole dataset was initially shuffled and divided into an 8:2 ratio for training and testing, respectively. During the training phase, the training dataset was further divided into five equal subsets to facilitate five-fold cross-validation. In this process, each of the five subsets, in turn, served as a validation set while the remaining four subsets were used for training. This process aided in parameter tuning, identifying the optimal model, and enhancing the reliability and generalizability of the predictive models. Each model was constructed through the Scikit-learn framework. The main optimized hyperparameters for each model were set as follows, which was operated by grid search (Ahmad et al., 2022) from different combinations of parameters: SVR (rbf kernel, *c* = 5); KNN (*k* = 8); RF (n_trees = 200, max_depth = 12); DT (max_depth = 9); MLP (max_iter = 350). The detailed performance is shown in Table 1.

**Table 1:** Evaluation results for five machine learning models.

	**SVR**	**KNN**	**RF**	**DT**	**MLP**
RMSE	9.217	9.495	**8.124**	10.469	11.327
MAE	6.954	7.227	**6.023**	7.814	8.588
*R* ^2^	0.277	0.281	**0.594**	0.099	0.237
SI (%)	13.14	11.52	**9.72**	9.98	13.60

Bold values indicate that the metrics from the corresponding method get the best performance.

The results presented in the table confirm the feasibility of utilizing smart home indicators for heart rate prediction, as demonstrated by the performance of the five machine learning models with a mean MAE value of 7.321. Among the evaluated models, RF achieved the best performance in terms of MAE, with a value of 6.023. This indicates that RF achieved the lowest average prediction error for the heart rate value compared to the other models. Additionally, RF also exhibited the lowest RMSE (8.124) and the highest *R*^2^ value (0.594), indicating superior accuracy and goodness of fit. KNN and SVR also performed reasonably well, with RMSE values of 9.495 and 9.217, respectively. Both models showed similar MAE values, with KNN at 7.227 and SVR at 6.954 suggesting comparable average prediction errors and accuracy. In contrast, DT and MLP exhibited higher RMSE and MAE values, indicating greater prediction errors compared to the other models. DT achieved the lowest *R*^2^ value of 0.099, indicating a weaker fit between predicted and actual heart rate values. MLP also had a relatively low *R*^2^ value of 0.237, suggesting a moderate fit to the actual data. When considering the SI, which provides an overall measure of model performance, RF achieved the lowest value of 9.72%, followed by DT (9.98%), KNN (11.52%), SVR (13.14%), and MLP (13.60%). It is worth noting that a SI below 10% is considered good for model performance in regression tasks (Oyeleye et al., 2022). Based on their performance, RF outperformed the other models, demonstrating the lowest prediction deviation, highest accuracy, and the lowest SI, while KNN and SVR also showed competitive performance. DT and MLP, on the other hand, exhibited relatively weaker performance compared to the other models. Overall, the evaluation results indicates that that leveraging smart home indicators with machine learning models can be a promising approach for heart rate prediction.

#### 4.2.2 Different phases and scenarios

**Figure 5:**
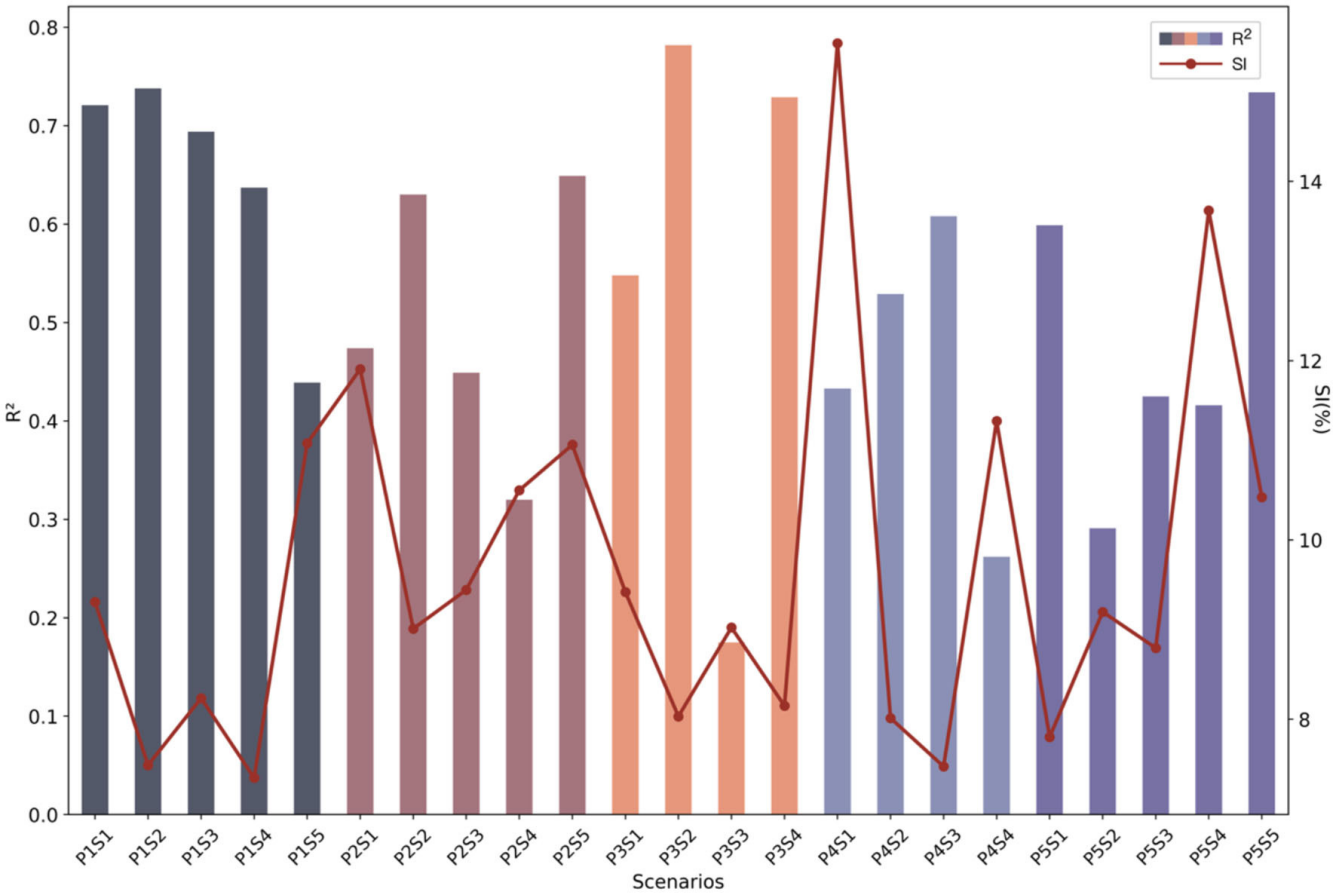
RF performance for different scenarios.

To gain insight into the prediction performance across different phases and scenarios, we conducted a separate evaluation for each of the 23 predefined activity scenarios (Box 1). The results, obtained using the RF model, are presented in Figure 5 and Table 2. Figure 5 combines the SI line chart with the *R*^2^ bar chart, enabling a comprehensive comparison of model's performance across different phases and scenarios, and facilitating an understanding of the relationship between *R*^2^ and SI for each scenario. Table 2 provides detailed results for all evaluation metrics, serving as reference for interpretation. Results from them demonstrate that the machine learning models, particularly RF model, exhibited moderate to good prediction accuracy for heart rate values in various orientated phases and scenarios.

**Table 2:** Detailed results on phases and scenarios based on RF.

		**Scenario1**	**Scenario2**	**Scenario3**	**Scenario4**	**Scenario5**	**Avg**
Phase 1	RMSE	7.974	5.987	6.583	6.154	9.560	7.252
	MAE	6.152	4.560	4.447	4.894	6.643	5.339
	*R* ^2^	0.721	0.738	0.694	0.637	0.439	0.646
	SI (%)	9.31	7.48	8.23	7.34	11.08	8.68
Phase 2	RMSE	9.811	7.414	7.923	8.856	9.473	8.695
	MAE	7.051	5.214	5.682	6.048	7.352	6.269
	*R* ^2^	0.474	0.630	0.449	0.320	0.649	0.504
	SI (%)	11.90	9.01	9.44	10.55	11.06	10.39
Phase 3	RMSE	7.369	7.202	7.596	6.413		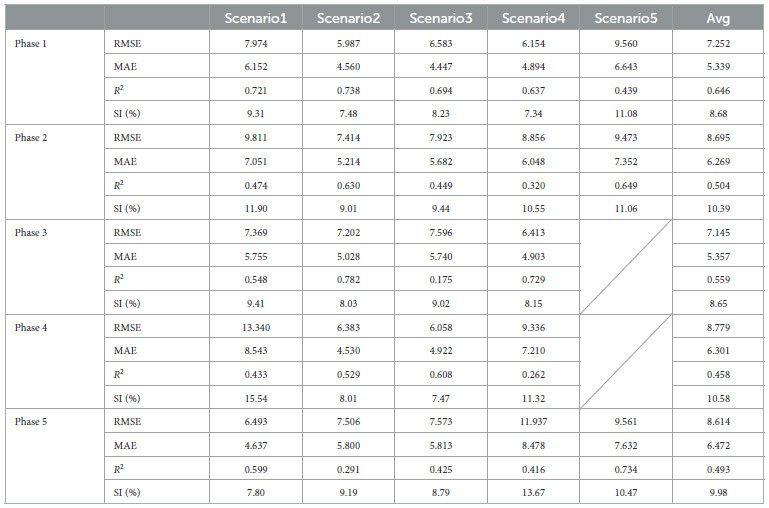
	MAE	5.755	5.028	5.740	4.903		5.357
	*R* ^2^	0.548	0.782	0.175	0.729		0.559
	SI (%)	9.41	8.03	9.02	8.15		8.65
Phase 4	RMSE	13.340	6.383	6.058	9.336		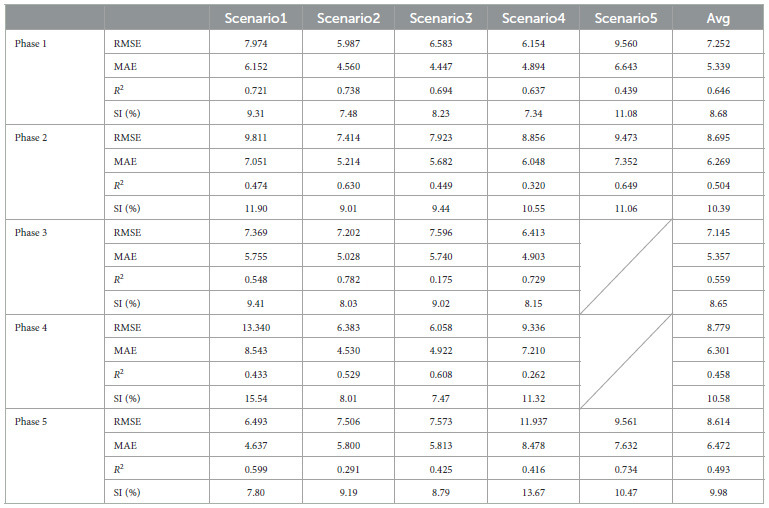
	MAE	8.543	4.530	4.922	7.210		6.301
	*R* ^2^	0.433	0.529	0.608	0.262		0.458
	SI (%)	15.54	8.01	7.47	11.32		10.58
Phase 5	RMSE	6.493	7.506	7.573	11.937	9.561	8.614
	MAE	4.637	5.800	5.813	8.478	7.632	6.472
	*R* ^2^	0.599	0.291	0.425	0.416	0.734	0.493
	SI (%)	7.80	9.19	8.79	13.67	10.47	9.98

##### 4.2.2.1 Phases

The analysis of evaluation results provided valuable insights into performance of the RF model across different phases. The model demonstrated its strong performance in Phase 3, which includes movement-oriented activities. In this phase, the RF model achieved the lowest RMSE (7.145) and the second lowest MAE (5.375), indicating higher precision and accuracy in predicting heart rate values for such activities. In contrast, Phase 4, representing basic ADLs, had the highest RMSE value of 8.779 among the phases, followed by Phase 2 (8.695). Phase 5 exhibited the highest MAE value of 6.472, followed by Phase 4 (6.301). Despite this, the RMSE and MAE values across all phases are within a reasonable range, indicating a generally acceptable level of prediction accuracy.

In terms of the *R*^2^ scores, three out of five phases (Phase 1, Phase 2, and Phase 3) achieved *R*^2^ scores consistently exceeding 0.5, while the other two phases approached 0.5. Particularly, Phase 1 achieved the highest *R*^2^ score (0.646), closely followed by Phase 3 with a *R*^2^ score of 0.559. Similarly, when considering SI values, three out of five phases (Phase 1, Phase 3, Phase 5) achieved SI values below 10% with the remaining two slightly exceeding this threshold. The lowest SI is observed in Phase 3 with 8.65%, closely followed by Phase 1 at 8.68%.

##### 4.2.2.2 Scenarios

Further analysis of individual scenarios within each phase revealed notable variations between activities. Among the different scenarios, Phase 1 Scenario 2 (P1S2) involving eating dinner exhibited the lowest RMSE (5.987), closely followed by P4S3 which pertains to meal preparation. On the other hand, the highest RMSE was observed on P4S1, associated with tasks of cleaning rooms. Additionally, the model demonstrated strong performance in terms of MAE during Phase 3 with minimal variance ranging from 4.903 to 5.755. Notably, P3S2 (morning exercise) and P3S4 (wandering in rooms) achieved low MAE values of 4.903 and 5.028, respectively. Furthermore, P1S3 obtained the lowest MAE with 4.447 while P4S1 recorded the highest MAE with a value of 8.543.

In terms of *R*^2^ scores. The highest *R*^2^ score of 0.78 was observed for the morning exercise scenario (P3S2). Similarly, the scenario of wandering in rooms (P3S4) exhibited a relatively high *R*^2^ score. However, the scenario of taking a shower (P3S3), which lacked sensor coverage in the shower area, recorded the lowest *R*^2^ score of 0.17. This disparity highlights the impact of sensor availability on the predictive accuracy of heart rate during specific activities. For SI score, which measures the variability of the predicted values around the true values, the RF model showed good performance with a majority of the scenarios having an SI score of < 10%. However, some scenarios had higher SI scores, with the highest being 15.54% for cleaning rooms (P4S1). Moreover, model performed moderately well in Phase 1, with the lowest SI score of 7.34% observed for cooking (P1S4). Even though taking shower had the lowest *R*^2^ score, its SI score was only 9.02%.

#### 4.2.3 Sensor and feature importance

**Figure 6:**
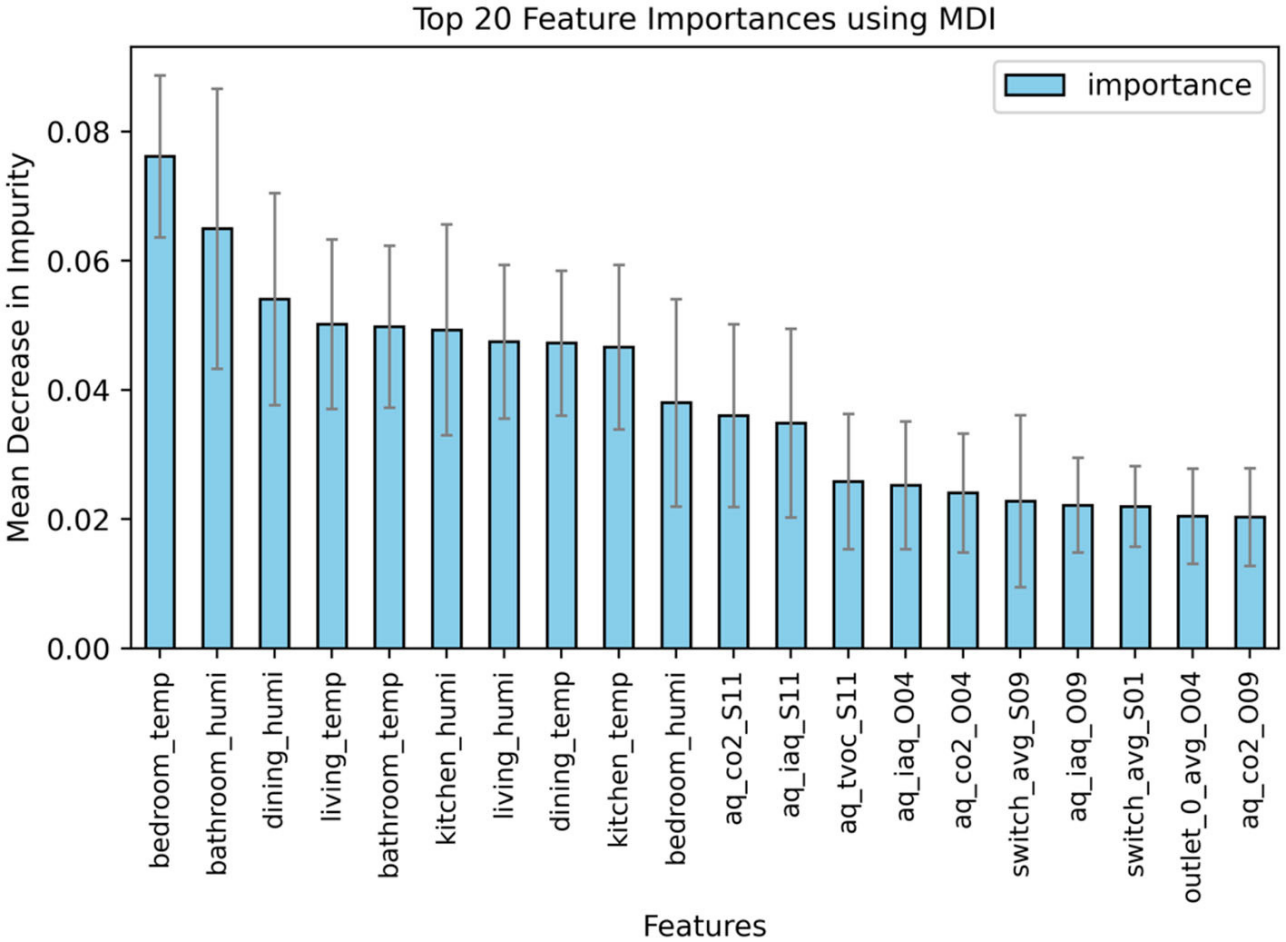
Feature importance using MDI based on RF model.

To determine which sensors and features most significantly influence heart rate prediction, based on RF model, we utilized the Mean Decrease in Impurity (MDI) metric to quantify the relative importance of each feature. We identified the top 20 features that significantly impact heart rate prediction, as illustrated in Figure 6.

Figure 6 illustrates that features related to temperature and humidity in all five rooms prominently dominate the feature importance hierarchy, with the top 10 spots occupied by these variables. Specifically, temperature readings in the bedroom is the most influential feature, achieving the highest MDI value of 0.76. The humidity level in the bathroom follows closely, marked as the second most critical feature with an MDI value of 0.65.

Subsequently, air quality-related features, as measured by relevant sensors, including CO_2_ levels, Air Quality Index, and Total Volatile Organic Compounds, occupy seven of the next 10 spots in feature importance. The sensors monitoring these parameters were strategically positioned in key home areas such as the living room (sensor O04), bedroom (sensor O09), and bathroom (sensor S11).

Moreover, the analysis reveals that features associated with the use patterns of switches and electrical outlets at specific locations also contribute significantly to the model's predictive performance. For instance, switch sensors S01 in the living room and S09 outside the bathroom, which were frequently interacted with by participants, proved to be influential.

These findings highlight the critical role of sensors monitoring environmental conditions like temperature, humidity, and air quality in impacting the model accuracy for heart rate prediction. This is consistent with the results from Ren et al. (2011), which suggest a strong correlation between these environmental factors and heart rate response, implicating their impact on cardiovascular health.

## 5 Discussion

### 5.1 Principal findings

The principal findings of this study demonstrate the potential and feasibility of using machine learning methods in predicting HR based on AAL technologies. All machine learning models employed in the study achieved favorable prediction results, with the RF model standing out as particularly noteworthy. Its capabilities in capturing the relationship between daily living activities and heart rate responses can make it a promising tool for accurate heart rate prediction in the future.

Furthermore, the study suggested that the performance of the RF model varied depending on the specific daily living activities being performed. A common knowledge we can take from the results is that activities that involved more indoor movement and triggered multiple sensors within the smart home environment resulted in higher prediction performance. On the other hand, activities involving static positions or posture changes showed comparatively lower prediction performance. These findings emphasize the importance of constructing an AAL environment with an adequate number of smart home devices, enhancing higher prediction performance.

### 5.2 Public health perspective

The findings of this research have valuable implications for public health. Accurately predicting heart rate based on contactless smart home technologies during individuals' daily living activities enables healthcare providers and public health agencies to gain a comprehensive understanding of an individual's cardiovascular health profile. This valuable information can inform the implementation of personalized interventions, preventive measures, and lifestyle modifications to mitigate the risk of cardiovascular diseases and improve overall health outcomes. By monitoring heart rate fluctuations using non-intrusive AAL technology, public agencies can detect early signs of cardiovascular issues, provide timely interventions, and allocate resources more efficiently to address cardiovascular health challenges at both the individual and population levels, reducing the burden of chronic diseases and improving the wellbeing of older adults.

### 5.3 Novelty

This study contributes to the existing research by addressing the limitations of previous studies that primarily focus on wearable devices and IoT technologies, which may be burden for some older adults. Unlike other studies (Al Rasyid et al., 2015; David Chung Hu et al., 2018; Ali et al., 2019; Xiao et al., 2020), this research investigates the feasibility of utilizing contactless smart home technologies alone, without the need for additional wearable devices, making it more practical for daily living contexts. Even when compared to studies that also monitor heart rate in a contactless manner (Adib et al., 2015; Kumar et al., 2022; Liu et al., 2022; Alnaggar et al., 2023), this study demonstrates distinct advantages. Firstly, it is not limited by large body movements since it does not rely on detecting chest or other physiological motions. This makes the proposed solution particularly effective in daily living scenarios. Secondly, there are fewer privacy concerns as it does not require visual monitoring. Lastly, the proposed solution exhibits environmental robustness, overcoming challenges such as high-frequency noise and variable lighting conditions that can affect other systems. By leveraging AI techniques, this study provides an advanced approach that unobtrusively monitors heart rate within an AAL environment, thus bridging the existing gap in research. The integration of contactless smart home technologies and machine learning algorithms enables heart rate monitoring without relying on user compliance, overcoming the challenges associated with wearable devices.

### 5.4 Limitations and future work

It is crucial to acknowledge the limitation of this study, which is the age range of the sample population. The participants in our study primarily consisted of young individuals, and it is important to recognize that physiological responses and behaviors may differ among different age groups, causing the bias in studies results. Another limitation is the fixed order for ADLs in the experiment, which may not accurately reflect real-world scenarios and some previous activities could potentially impact HR response during subsequent activities. Furthermore, the dataset for our analysis was limited to evaluable data from only 28 subjects, a sample size that is not sufficient for a comprehensive analysis of robustness. While these data provide useful preliminary insights, this limitation in sample size constrains the generalizability of our findings.

Despite these limitations, our study still represents a notable advancement in utilizing SHTs as a valuable tool for healthcare monitoring, demonstrating the feasibility and potential effectiveness of using contactless smart home technology for heart rate monitoring. To further strengthen the validity and applicability of our findings, future research will address these limitations by evaluating with a larger group of participants and including a more representative sample of older adult participants. In addition, further study will be conducted in real-world in an uncontrolled settings to evaluate impacts of numbers and locations of smart home sensors to prediction performance, exploring optimal configuration of sensors in a AAL environment.

## 6 Conclusions

The results of this study highlight the promising potential of utilizing contactless SHTs coupled with machine learning techniques for accurate heart rate prediction during individuals' daily living activities within AAL environments. Integrating contactless SHTs into individuals' living environments facilitates a convenient and unobtrusive method for tracking variations in heart rate during different activities. This approach not only addresses common issues such as user compliance and sensor burden found in traditional systems but also assist individuals to discern the diverse impacts of various activities on their heart rate, fostering a more engaged and informed approach to personal health monitoring. This integration of technology into the daily lives of older adults enables unobtrusive monitoring of their cardiovascular health, providing valuable insights for healthcare providers and public health officials. The approach can be further integrated into the UbiLab AAL Data Ecosystem, providing comprehensive indoor healthcare surveillance for older adult population.

## Data availability statement

The raw data supporting the conclusions of this article will be made available by the authors, without undue reservation.

## Ethics statement

The studies involving humans were approved by Research Ethics Office of the University of Waterloo. The studies were conducted in accordance with the local legislation and institutional requirements. The participants provided their written informed consent to participate in this study.

## Author contributions

KW: Data curation, Formal analysis, Methodology, Validation, Writing—original draft, Writing—review & editing. SC: Supervision, Writing—review & editing. JK: Writing—original draft, Writing—review & editing. MG: Writing—review & editing. ZAB: Writing—review & editing. PM: Project administration, Supervision, Writing—review & editing.

## Appendix

**Table A1:** Features table.

**Feature name**	**Description**
outlet_0_on_O01-O18	18 features Binary data reflecting the on/off states of the upper ports as captured by outlet sensors designated as O01 to O18
outlet_1_on_O01-O18	18 features Binary data reflecting the on/off states of the lower ports as captured by outlet sensors designated as O01 to O18
outlet_0_avg_O01-O18	18 features Continuous data reflecting the energy usage of the upper ports as captured by outlet sensors designated as O01 to O18
outlet_1_avg_O01-O18	18 features Continuous data reflecting the energy usage of the lower ports as captured by outlet sensors designated as O01 to O18
switch_avg_S01-S11	11 features Continuous reflecting the energy usage of switch sensors designated as S01 to S11
living_temp	10 features Measurement of temperature and humidity across five rooms
living_humi	
kitchen_temp	
kitchen_humi	
dining_temp	
dining_humi	
bedroom_temp	
bedroom_humi	
bathroom_temp	
bathroom_humi	
motion_01-23	23 features Binary data reflecting the occupancy states from 23 passive infrared sensors
aq_co2_O04	9 features Measurement of air quality related parameters from 3 environmental sensors
aq_iaq_O04	
aq_tvoc_O04	
aq_co2_O09	
aq_iaq_O09	
aq_tvoc_O09	
aq_co2_S11	
aq_iaq_ S11	
aq_tvoc_S11	
door_contact_01-12	12 features Binary data reflecting the door open/close states from 12 door contact sensors
